# Monitoring of Peripheral Blood Leukocytes and Plasma Samples: A Pilot Study to Examine Treatment Response to Leflunomide in Rheumatoid Arthritis

**DOI:** 10.3390/ph14020106

**Published:** 2021-01-29

**Authors:** João F. S. Rodrigues, Liziane C. M. da Silva, Leia Cardoso-Sousa, Douglas Carvalho Caixeta, Debora D. Lückemeyer, Alisson S. Henrique, Jaqueline P. Pontes, Lycia M. G. da Silva, Juliana S. S. Macedo, Pedro S. Carvalho Júnior, Cristiane Silva e Silva, Mahiba M. R. S. Martins, Valério Monteiro-Neto, Marcos A. G. Grisotto, Anita M. R. Fernandes, Juliano Ferreira, João B. Calixto, Robinson Sabino-Silva, Elizabeth S. Fernandes

**Affiliations:** 1 Programa de Pós-Graduação, Universidade CEUMA, São Luís 65075-120, Brazil; joaofranciscosr@hotmail.com (J.F.S.R.); cristiane.figueiredo@ceuma.br (C.S.e.S.); 2 Instituto de Pesquisa Pelé Pequeno Príncipe, Curitiba 80250-060, Brazil; lizianecms@gmail.com; 3 Programa de Pós-Graduação em Biotecnologia Aplicada à Saúde da Criança e do Adolescente, Faculdades Pequeno Príncipe, Curitiba 80230-020, Brazil; 4 Instituto de Ciências Biomédicas, Universidade Federal de Uberlândia, Uberlândia 38400-902, Brazil; leiacardosos92@gmail.com (L.C.-S.); caixetadoug@gmail.com (D.C.C.); robinsonsabino@gmail.com (R.S.-S.); 5 Departamento de Farmacologia, Universidade Federal de Santa Catarina, Florianópolis 88040-900, Brazil; debora_dl@hotmail.com (D.D.L.); juliano.ferreira@ufsc.br (J.F.); 6 Mestrado em Computação Aplicada, Universidade do Vale do Itajaí, Itajaí, São José 88102-700, Brazil; ali.steffens@gmail.com (A.S.H.); anita.fernandes@univali.br (A.M.R.F.); 7 Programa de Pós-Graduação em Ciências da Saúde, Universidade Federal do Maranhão, São Luís 65080-805, Brazil; jaquelinepessoap@gmail.com (J.P.P.); valerio.monteiro@ufma.br (V.M.-N.); 8 Farmácia Estadual de Medicamentos Especializados, Secretaria Estadual de Saúde do Maranhão, São Luís 65010-650, Brazil; lycia_guimaraes@hotmail.com.br (L.M.G.d.S.); juliana_sandes@hotmail.com (J.S.S.M.); pedrosatiro@yahoo.com.br (P.S.C.J.); 9 Centro de Especialidades Médicas Vinhais, São Luís 65075-180, Brazil; mmmahiba@hotmail.com; 10 Spedali Civili di Brescia, DSCS-c/o U.O. Reumatologia e Immunologia Clinica, Brescia, 25123 Lombardia, Italy; marcos.grisotto@gmail.com; 11 Centro de Inovação e Ensaios Pré-Clínicos-CIEnP, Florianópolis 88056-000, Brazil; joao.calixto@cienp.org.br

**Keywords:** rheumatoid arthritis, treatment response, leflunomide, peripheral blood cells, ATR-FTIR

## Abstract

Rheumatoid arthritis (RA) is a painful inflammatory disease of the joints which affects a considerable proportion of the world population, mostly women. If not adequately treated, RA patients can become permanently disabled. Importantly, not all the patients respond to the available anti-rheumatic therapies, which also present diverse side effects. In this context, monitoring of treatment response is pivotal to avoid unnecessary side effects and costs towards an ineffective therapy. Herein, we performed a pilot study to investigate the potential use of flow cytometry and attenuated total reflection–Fourier transform infrared (ATR-FTIR) spectroscopy as measures to identify responders and non-responders to leflunomide, a disease-modifying drug used in the treatment of RA patients. The evaluation of peripheral blood CD62L^+^ polymorphonuclear cell numbers and ATR-FTIR vibrational modes in plasma were able to discriminate responders to leflunomide (LFN) three-months after therapy has started. Overall, the results indicate that both flow cytometry and ATR-FTIR can potentially be employed as additional measures to monitor early treatment response to LFN in RA patients.

## 1. Introduction

Rheumatoid arthritis (RA) is a painful inflammatory disease of the joints which affects ~0.5–1% of the world population, mostly women. If not treated, RA patients can become permanently disabled due to the intense articular pain, stiffness and oedema resulting from chronic joint inflammation. In addition to the joint pathology, RA patients also frequently suffer from systemic alterations such as anemia. Despite its morbidity and prevalence, the currently available anti-rheumatic therapy does not halt disease progression. It has also been reported that not all patients respond to such therapies.

International guidelines for early RA recommend treatment to be started as soon as possible with disease-modifying drugs (DMARDs). Methotrexate is used as the first treatment strategy for RA; however, other DMARDs such as leflunomide (LFN) have been largely prescribed to those with contraindication to methotrexate [1]. LFN acts on T cells blocking their proliferation via inhibition of pyrimidine synthesis and on peripheral blood mononuclear cells by reducing their activation [2,3]. Despite its efficacy, it is estimated that 5–10% of RA patients do not improve with LFN following a six-month treatment [4]. Increases in dosage schemes may result in superior but not always ideal efficacy, and also in greater adverse effects [5,6,7]. In the case of LFN discontinuation, this therapy is replaced by another antirheumatic drug and the patient is then, submitted to another trial course, with no guarantees of successful therapy.

Ideally, the response of patients to RA therapy should be monitored as soon as treatment initiates as it would avoid suffering and costs towards an ineffective therapy. The determination of clinical improvement or response to RA treatment is complex and several RA disease activity measures have been proposed for this purpose. These are suggested to be variable in performance and to not always be reliable and feasible for ordinary use [8]. The American College of Rheumatology recently recommended a list of preferred measures [8]; however, these should not be considered mandatory and should be revised as novel measures are developed.

As inflammation is a hallmark of RA, markers of response to LFN have been investigated in inflammatory cells, especially in peripheral blood cell samples [9,10,11,12]. Indeed, polymorphisms of dihydroorotate dehydrogenase and its increased expression in B-lymphocytes were associated with higher resistance to treatment with LFN [9,11]. Similarly, LFN-resistant subjects exhibit increased levels of the cell membrane transporter ABCG2 on their synovial macrophages [12]. In this context, cell counts and activation profiles of peripheral blood leukocytes such as polymorphonuclear (PMN) and mononuclear cells may be useful to monitor active RA.

Despite the advances in the field, there is still much to learn about the prediction of response to LFN in RA. Attenuated total reflection–Fourier transform infrared (ATR-FTIR) spectroscopy has been used as a sustainable, reagent-free, and rapid tool to obtain molecular fingerprints of biological samples such as plasma, with minimal sample preparation [13,14]. This is particularly interesting considering the ability of ATR-FTIR to discriminate between normal and pathological conditions, indicating its potential clinical use for diagnosing, screening, or monitoring of the progression/regression of various diseases [14,15,16,17,18,19]. Herein, we used ATR-FTIR and flow cytometry to identify novel potential peripheral biomarkers of response to LFN in RA patients. The results indicate these tools may be useful complementary measures to examine treatment response in patients with RA.

## 2. Results

### 2.1. Subject Characteristics

**Figure 1:**
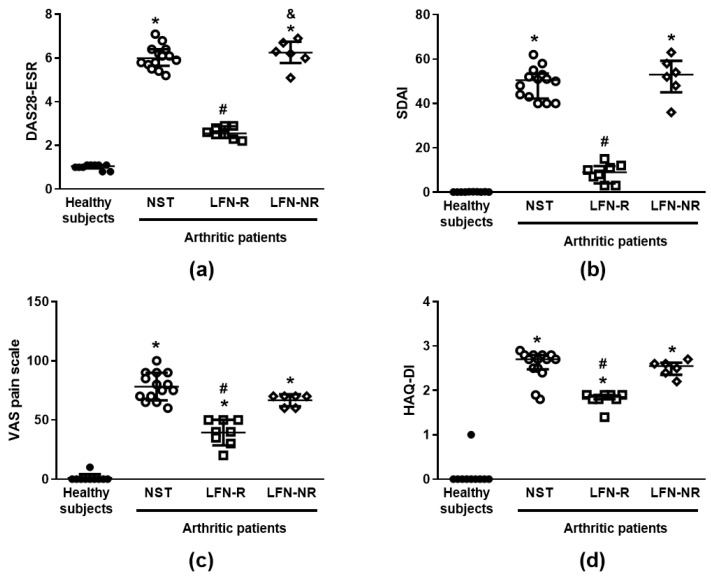
Disease activity indexes. (**a**) Disease Activity Score-28 for Rheumatoid Arthritis with erythrocyte sedimentation rate (ESR) (DAS28-ESR), (**b**) Simplified Disease Activity Index for Rheumatoid Arthritis (SDAI), (**c**) visual analog scale (VAS) and (**d**) Stanford Health Questionnaire–disability index (HAQ-DI) in patients with rheumatoid arthritis (RA) treated with leflunomide (LFN; 20 mg/day, *per os*). The scores were recorded from patients recently diagnosed with RA but not yet receiving specific treatment with anti-rheumatic drugs prior to (non-specific therapy (NST) group; *n* = 14) and three-months after LFN treatment. Patients presenting at least a 50% reduction of the disease activity indexes and at least a 30% reduction of the pain and/or disability scores following treatment were considered as responders to LFN (LFN-R; *n* = 8). Those with high disease activity indexes (DAS28-ESR and SDAI) and with less than 30% reduction in the VAS and HAQ-DI scores after three-months of LFN treatment were considered as non-responders (LFN-NR; *n* = 6). Healthy subjects (*n* = 10) were used as controls. Data are expressed as scatter plots with mean ± SD (panel **c**) or median and 25th–75th percentile (IQR; panels **a**, **b** and **d**). ∗ *p* < 0.05, differs from healthy subjects; ^#^ *p* < 0.05, differs from prior to therapy (NST group); ^&^ *p* < 0.05, differs from the LFN-R group.

Data depicted in Table 1 shows that the arthritic population which enrolled (patients who had not yet received specific therapy; NST group) and continued throughout the study, primarily consisted of women above 50 years of age (93%; 13 out of 14 participants). The majority of the patients had the disease for less than 10 years (86%; 12 out of 14 participants). Prior to LFN treatment, most of them presented swollen joints (86%; 12 out of 14 participants) and 21% (3 out of 14 participants) had lumps or deformities (Table 1). All RA patients exhibited high disease activity (Disease Activity Score-28 for Rheumatoid Arthritis with erythrocyte sedimentation rate(ESR) (DAS28-ESR) and Simplified Disease Activity Index for Rheumatoid Arthritis (SDAI)) indexes; Table 1 and Figure 1a,b). They also presented with enhanced pain sensitivity and disability, denoted by the visual analog scale (VAS) and the Stanford Health Assessment Questionnaire (HAQ)-disability index (HAQ-DI) scores, in comparison with healthy subjects (Figure 1c,d). Eight women and two men composed the population of healthy subjects, with an average age of 33.3 ± 2.8 years.

Following treatment, 57% of the patients (8 out of 14) were found to respond to LFN (responder group; LFN-R) as they presented lower DAS28-ESR and SDAI indexes (Table 1 and Figure 1a,b) which were compatible with lower disease activity or remission [20,21]. Indeed, considering the DAS28-ESR, 50% of the LFN-R patients achieved remission and 50% a low disease activity. Data from the SDAI indicate that 25% of the patients of the LFN-R group achieved remission, whilst low and moderate disease activities were observed for 50% and 25% patients, respectively. The same patients presented less pain and disability (Figure 1c,d), and lower circulating levels of C-reactive protein and rheumatoid factor than prior to treatment (Table 1). Accordingly, fewer patients of the LFN-R group exhibited swollen joints (Table 1). On the other hand, non-responders (LFN-NR) consisted of 43% of the assessed patients (6 out of 14; Table 1 and Figure 1a–d). The levels of C-reactive protein, rheumatoid factor, DAS28-ESR, SDAI, as well as the pain thresholds and disability scores observed for the LFN-NR group were similar to those recorded prior to treatment (Table 1 and Figure 1a–d). All LFN-NR patients presented swollen joints (Table 1).

### 2.2. Circulating Polymorphonuclear and Mononuclear Cells

**Figure 2:**
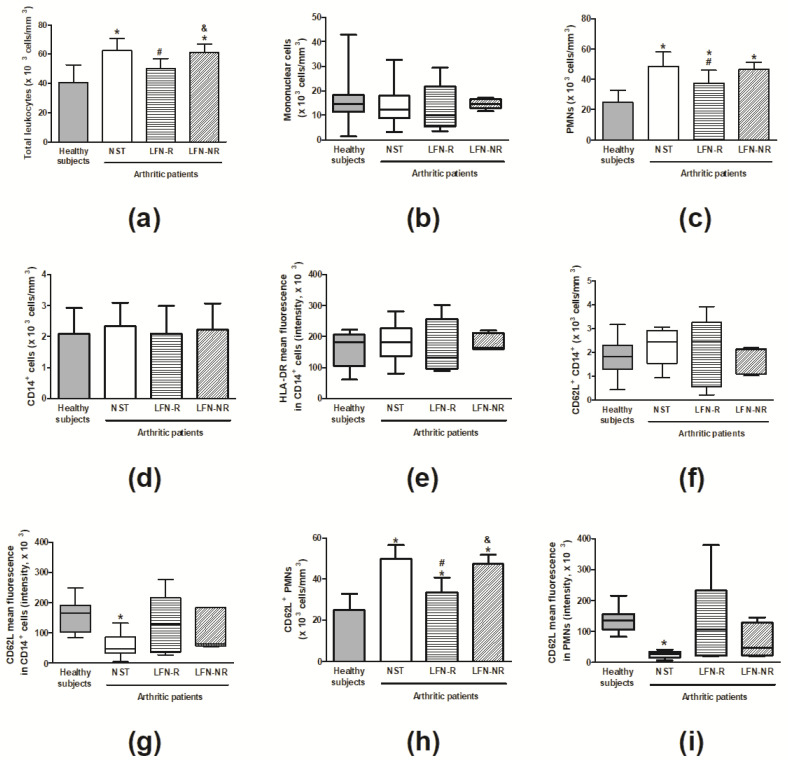
Flow cytometry analysis of peripheral blood cells. (**a**) Total leukocytes, (**b**) mononuclear and (**c**) polymorphonuclear (PMN) cells. Numbers of (**d**) CD14^+^ and (**f**) CD62L^+^CD14^+^ cells, and (**e**) HLA-DR and (**g**) CD62L mean fluorescence in CD14^+^ cells. (**h**) CD62L^+^PMNs and (**i**) CD62L mean fluorescence in PMNs. Peripheral blood cells were isolated from patients recently diagnosed with rheumatoid arthritis (RA) but not yet receiving specific treatment with anti-rheumatic drugs, prior to leflunomide (LFN; 20 mg/day, *per os*) treatment (NST group; *n* = 14). Three-months following treatment, samples were collected from both responders (LFN-R; n = 8) and non-responders (LFN-NR; n = 6) to LFN. Healthy subjects (n = 10) were used as controls. Data are expressed as mean ± SD (panels **a**, **c**, **d** and **h**) or median and minimum-maximum values (panels **b**, **e**–**g** and **i**). ∗ *p* < 0.05, differs from healthy subjects; ^#^ *p* < 0.05, differs from prior to therapy (NST group); ^&^ *p* < 0.05, differs from the LFN-R group.

Data depicted in Figure 2 indicates the total counts of leukocytes, and the numbers of PMN and mononuclear cells of patients with RA and healthy subjects. Before treatment, patients with RA (NST group) presented with increased numbers of total leukocytes and PMNs, with no significant differences in mononuclear cells (Figure 2a–c). Following therapy, LFN-R but not LFN-NR patients, had reduced counts of these cell populations in comparison with before treatment (NST) counts (Figure 2a,c).

The markers of PMN and mononuclear cell activation CD62L and HLA-DR were analyzed. No differences in the numbers of CD14^+^ and in the expression of HLA-DR on CD14^+^ cells were observed between groups (Figure 2d,e). Although no differences were seen in the numbers of CD62L^+^CD14^+^ cells (Figure 2f) prior to LFN therapy, CD62L expression was markedly reduced on CD14^+^ and PMN cells of NST RA patients in comparison with healthy subjects (Figure 2g,i). These patients exhibited higher numbers of CD62L^+^PMNs (Figure 2h). Following treatment, the LFN-R group presented with diminished numbers and increased activation of these cells (Figure 2h,i).

**Figure 3:**
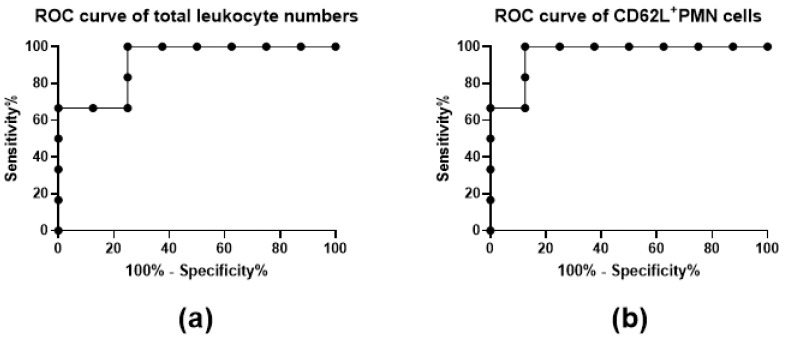
Receiver Operator Characteristic (ROC) curve analysis of (**a**) total leukocyte and (**b**) CD62L^+^PMN cell numbers to LFN-R and LFN-NR individuals. Samples were obtained from patients recently diagnosed with rheumatoid arthritis following three-month treatment with leflunomide (LFN; 20 mg/day, per os) (LFN group; *n* = 8; LFN-NR group; *n* = 6).

Receiver Operator Characteristic (ROC) curve analyses were performed to test the specificity and sensitivity of cell population measurements in discriminating between LFN-R and LFN-NR patients. As significant differences between the LFN-R and LFN-NR groups were only observed for total leukocyte and CD62L^+^PMN cell numbers (Figure 2a,h), ROC analyses were only carried out for these parameters. A sensitivity of 100% and a specificity of 75% was detected for the number of total leukocytes (*p* < 0.01; cut-off: 52,809 cells/mm^3^; area under the curve (AUC): 0.92) (Figure 3a). The sensitivity and specificity values for CD62L^+^PMNs were 100% and 88%, respectively (*p* < 0.01; cut-off: 40,082 cells/mm^3^; AUC: 0.96) (Figure 3b).

### 2.3. Spectral Analysis of Plasma by ATR-FTIR

**Figure 4:**
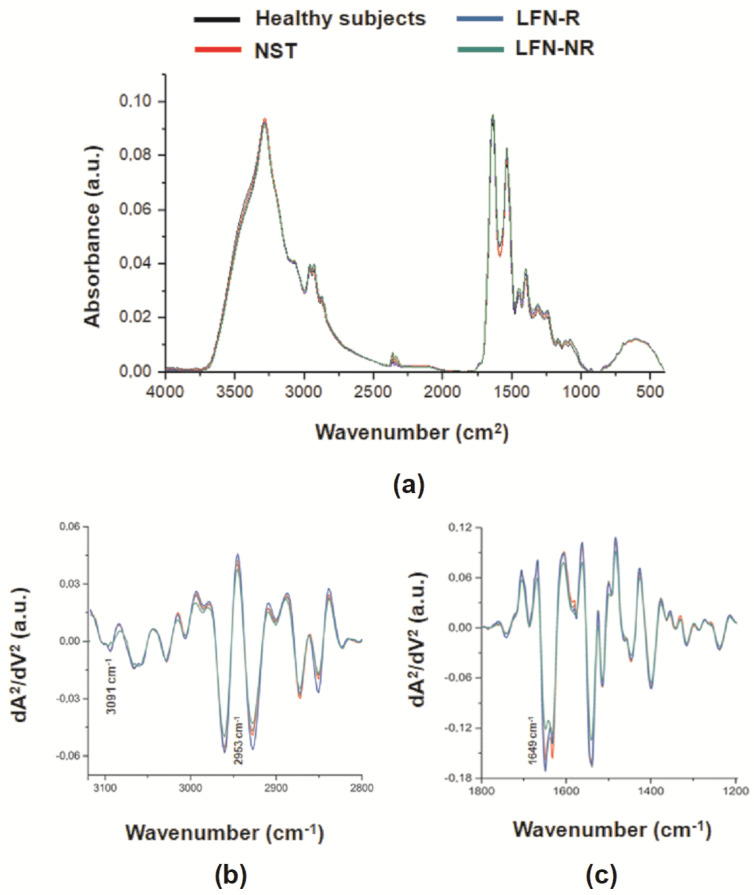
Representative spectral markers in plasma samples by FTIR analysis. Average of infrared spectrum (**a**) and representative spectral regions of lipids (**b**) and proteins (**c**) in plasma samples obtained from healthy subjects (*n* = 10), patients recently diagnosed with rheumatoid arthritis (RA) but not yet receiving specific treatment with anti-rheumatic drugs, prior to leflunomide (LFN; 20 mg/day, *per os*) treatment (NST group; *n* = 14). Three-months following treatment, samples were collected from both responders (LFN-R; *n* = 8) and non-responders (LFN-NR; *n* = 6) to LFN.

Figure 4a represents the average of infrared spectra of plasma samples obtained from RA patients prior to (NST) and after LFN treatment (LFN-R and LFN-NR patients) in comparison with those of healthy subjects. Each spectrum represents vibrational modes of molecules such as lipids, proteins, carbohydrates, glycoproteins and nucleic acid. To discriminate each vibrational mode we performed the second derivative analysis. Figure 4b displays the infrared vibrational modes in a region that represents mainly lipid molecules and Figure 4c represents the infrared vibrational modes in a region that contains proteic, carbohydrate, and nucleic acid molecules. Some vibrational bands of interest include: stretching vibration of C-O (amide I): β-sheet and α-helix from proteins (1649 cm^−1^; [15]), asymmetric stretching vibration of CH_3_: cholesterol esters, lipids (2953 cm^−1^; [22]), and stretching vibration of CH_2_: lipids (3091 cm^−1^; [23]).

**Figure 5:**
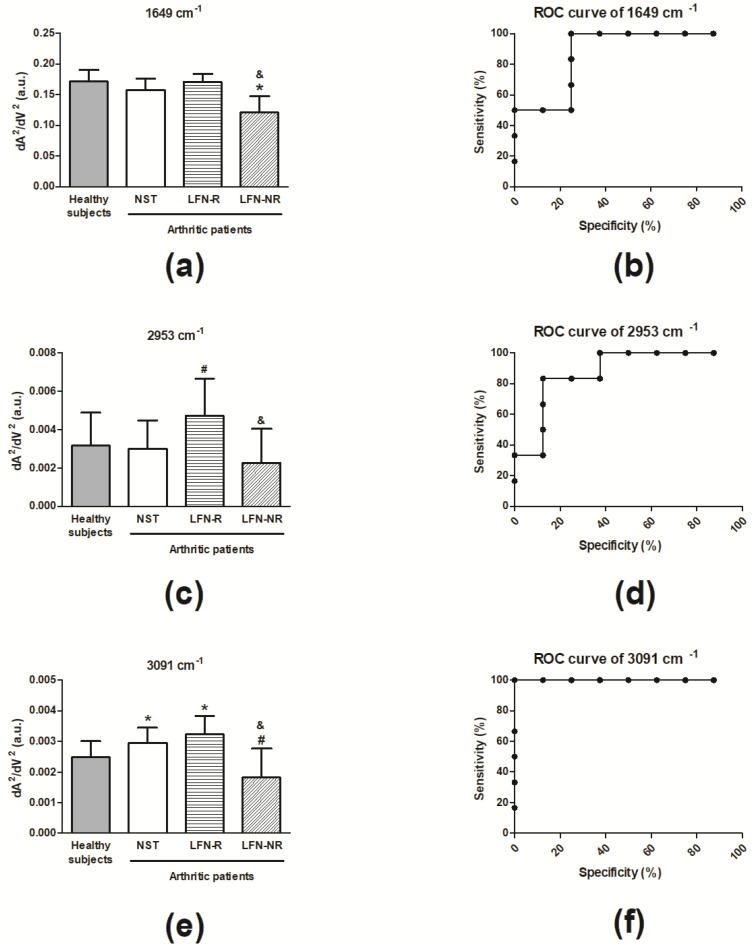
Vibrational mode values in plasma samples of rheumatoid arthritis (RA) patients. Vibrational modes at (**a**) 1649 cm^−1^, (**c**) 2953 cm^−1^ and (**e**) 3091 cm^−1^ from plasma samples of RA patients. ROC curve analysis of (**b**) 1649 cm^−1^, (**d**) 2953 cm^−1^ and (**f**) 3091 cm^−1^ to LFN-R and LFN-NR individuals. Samples were obtained from patients recently diagnosed with rheumatoid arthritis (RA) but not yet receiving specific treatment with anti-rheumatic drugs, prior to leflunomide (LFN; 20 mg/day, *per os*) treatment (NST group; *n* = 14). Three-months following treatment, samples were collected from both responders (LFN-R; *n* = 8) and non-responders (LFN-NR; *n* = 6) to LFN. Healthy subjects (*n* = 10) were used as controls. Data are expressed as mean ± SD (panels **a**, **c** and **e**). ∗ *p* < 0.05, differs from healthy subjects; ^#^ *p* < 0.05, differs from the NST group; ^&^ *p* < 0.05, differs from the LFN-R group.

Prior to LFN therapy, RA patients exhibited an increase (*p* < 0.05) of the 3091 cm^−1^ vibrational mode in comparison with healthy subjects (Figure 5e), and the vibrational modes at 1649 cm^−1^ and 2953 cm^−1^ were similar (*p* > 0.05) in both groups (Figure 5a,c). An elevation of the 2953 cm^−1^ vibrational mode was observed only in LFN-R patients (Figure 5c), whilst non-responders (LFN-NR) presented with lower levels of 1649 cm^−1^ and 3091 cm^−1^ (Figure 5a,e).

ROC curve analyses were performed to test the specificity and sensitivity of these vibrational modes in distinguishing between LFN-R and LFN-NR patients. A sensitivity of 100% and a specificity of 75% was detected for 1649 cm^−1^ (*p* < 0.03; cut-off: 0.1708; area under the curve (AUC): 0.88) (Figure 5b). The sensitivity and specificity values for the vibrational mode at 2953 cm^−1^ were of 83% and 88%, respectively (*p* < 0.03; cut-off: 0.0035; AUC: 0.88) (Figure 5d). For the vibrational mode at 3091 cm^−1^, both the sensitivity and specificity values were of 100% (*p* < 0.002; cut-off: 0.0025; AUC: 1.00) (Figure 5f).

## 3. Discussion

Herein, we performed a pilot study to evaluate the potential applicability of ATR-FTIR spectroscopy of plasma samples and flow cytometry analysis of peripheral blood leukocytes to monitor treatment response to LFN in RA patients. As expected, the study population was composed mostly of women who presented joint swelling and had RA for less than 10 years. Prior to LFN treatment, RA patients exhibited high disease activity indexes (DAS28-ESR and SDAI), and increased pain and disability paralleled to elevated C-reactive protein and rheumatoid factor levels. They also exhibited augmented numbers of peripheral blood leukocytes (especially CD62L^+^PMN cells), and reduced expression of the L-selectin CD62L on CD14^+^ cells and PMNs. These results are in accordance with previous reports as leukocytes shed CD62L when they become activated, a process which has been linked to cell migration to inflamed sites [24,25].

A reduction in disease activity of at least 50% within the first three-months of treatment, increases the chance of achieving remission or a low disease activity [1,26,27]. In this context, it is important to highlight that although not all patients classified as responders in this study had achieved remission or low disease activity indexes after three-months of treatment, they all presented a reduction ≥50% in both the DAS28-ESR and SDAI, suggesting that therapy with LFN is likely to succeed in LFN-R patients. Following a three-month treatment with LFN, responders but not non-responders presented with lower DAS28-ESR and SDAI indexes, less pain and disability, as well as lower levels of C-reactive protein and rheumatoid factor. These changes were accompanied by reduced numbers of CD62L^+^PMNs. On the other hand, restoration of CD62L expression on CD14^+^ and PMN cells was noted in all patients following LFN treatment. LFN was previously shown to inhibit the chemotaxis of peripheral blood CD14^+^ cells [3] and PMNs [28] obtained from RA patients. These data indicate that the number of CD62L^+^PMNs rather than the level of CD62L expression on peripheral blood leukocytes might influence the response to LFN.

As LFN-R and LFN-NR individuals exhibited significant differences in regards to total leukocyte and CD62L^+^ PMN numbers, ROC analyses were performed in order to test the potential of these groups of cells to discriminate between responders and non-responders. A sensitivity of 100% was registered for both total leukocytes and CD62L^+^ PMN cells. Similar accuracies were observed for these cell populations, indicating a great potential for their use as discriminators of responders and non-responders to LFN treatment.

Although promising, the regular use of these flow cytometry measurements to monitor treatment response implicate additional costs towards equipment and reagents, as well as trained personnel. ATR-FTIR has emerged as a simple to operate and inexpensive tool, as it does not require reagents [29]; therefore, consisting of a sustainable and rapid monitoring platform with application in biological samples of individuals with chronic diseases including diabetes [14,18], cancer [15,30] and arthritic conditions [16,17]. Herein, we identified novel spectral parameters in plasma samples of RA patients that were able to discriminate LFN-R and LFN-NR individuals (1649 cm^−1^, 2953 cm^−1^ and 3091 cm^−1^). ROC curve analyses demonstrated that the vibrational mode at 3091 cm^−1^ presented the best sensitivity and specificity (100%), followed by 1649 cm^−1^ (100% sensitivity and 75% specificity), and 2953 cm^−1^ (83% sensitivity and 88% specificity).

These vibrational bands corresponded to amide I (1649 cm^−1^; [15] and functional groups related to lipids such as cholesterol esters (2953 cm^−1^ and 3091 cm^−1^; [22,23]. Herein, lower levels of the amide I band were observed in non-responders to LFN. Importantly, this band is the most frequently assessed region in studies of collagen structural integrity, and its reduction may indicate increased loss of collagen structures [31,32]. We also found that those not responding to LFN within the investigated time-frame had reduced levels of the 2953 cm^−1^ and 3091 cm^−1^ bands in comparison to responders to treatment. Of note, RA patients are suggested to present accelerated atherosclerosis despite the lower levels of low-density lipoprotein cholesterol detected in these individuals [33]. Loss of function of high-density lipoprotein cholesterol and increased cholesterol ester catabolism may be present in patients with active disease [33]. The data suggest that patients with worsen disease present lower levels of cholesterol in their circulation and this is associated with comorbidities such as those of the cardiovascular system. Interestingly, Shah and collaborators [34] reported that high levels of C-reactive protein are associated with low circulating levels of cholesterol lipids in patients with RA. Additionally, RA patients undergoing DMARD treatment presented an opposite lipid profile (i.e., enhanced lipid levels), which was associated with reduction of the inflammatory response. Taken together, all these pieces of evidence support the potential application of ART-FTIR for monitoring treatment response in RA patients through the vibrational modes identified in the present study.

## 4. Materials and Methods

### 4.1. Patients

**Figure 6:**
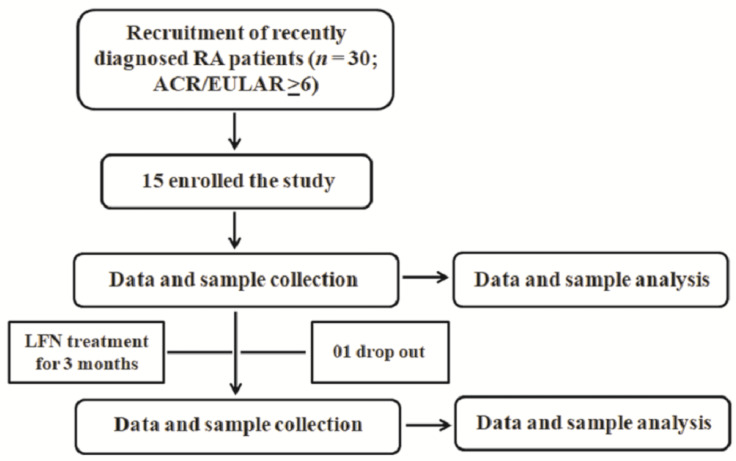
Study workflow. Patients recently diagnosed with RA presenting a score ≥6 on the 2010 ACR/EULAR Classification Criteria for Rheumatoid Arthritis, were recruited for participation in the study. All data and blood samples were collected from each rheumatoid arthritis patient at diagnosis (prior to treatment; NST group) and three-months after leflunomide (LFN; 20 mg/day, *per os*) treatment. Peripheral blood cells were analyzed by flow cytometry. C-Reactive protein and rheumatoid factor levels were assessed in plasma samples. Plasma samples were also analyzed by attenuated total reflection-Fourier transform infrared (ATR-FTIR) spectroscopy. Data such as gender, age, duration of disease, presence of swollen joints, lumps or deformities, as well as pain and disability scores were collected. Patients presenting with high disease activity scores (Disease Activity Score-28 for Rheumatoid Arthritis with ESR (DAS28-ESR) and Simplified Disease Activity Index for Rheumatoid Arthritis (SDAI) and less than 30% reduction in the visual analog scale (VAS) and Stanford Health Questionnaire-disability index (HAQ-DI) after three-months of LFN treatment were considered as non-responders to LFN. Patients presenting at least a 50% reduction of the disease activity indexes (DAS28-ESR and SDAI) and at least a 30% reduction of the pain and/or disability scores following treatment were considered as LFN-R.

A total of 30 patients (men and women) aged > 30 years, who had just been clinically diagnosed with RA and were about to start therapy with LFN, were recruited for participation in the study. Of those, 15 enrolled in the study and one patient dropped out after the first collection of data and samples. All patients were non-smokers, were naive for DMARDs but not corticoids, and had received no specific therapy for RA. At the beginning of the study, all patients presented synovitis symptoms (presence of joint pain, and swelling or lumps) and a score ≥6 on the 2010 ACR/EULAR Classification Criteria for Rheumatoid Arthritis, DAS28-ESR >5.1 and SDAI >26, as previously detailed [20,21,35]. Healthy subjects (*n* = 10; men and women aged >30 years) were used as controls and included non-smokers who had no history of recent infections, malignancy, or other autoimmune diseases and no present or previous use of DMARDs or experimental drugs. Individuals who were smokers, younger than 30-years of age, with a history of recent infections, malignancy, or other autoimmune diseases, and present or previous use of DMARDs, other specific anti-rheumatic therapies or experimental drugs were excluded from the study. All individuals were assessed for pain level and disability by the HAQ-DI (for review see: [36]) as previously described [37]. Five milliliters (ml) of peripheral blood were collected from each patient for separation of plasma and peripheral blood leukocytes, prior to and three-months after LFN therapy (20 mg/day, *per os*). All data and samples were collected prior to and three-months after LFN treatment. Patients presenting at least a 50% reduction of the disease activity indexes (DAS28-ESR and SDAI) and at least a 30% reduction of the pain and/or disability scores following treatment were considered as LFN-R. Those with high disease activity indexes (DAS28-ESR and SDAI) and with less than 30% reduction in the VAS and HAQ-DI scores after three-months of LFN treatment were considered as LFN-NR. The study was reviewed and approved by the Human Research Ethics Committee of the Universidade CEUMA (protocol number: 4.055.579). Informed consent was obtained from each participant. Figure 6 shows the workflow of analysis in RA patients.

### 4.2. Serum Rheumatoid Factor and C-Reactive Protein Levels

Rheumatoid factor and C-reactive protein levels were measured as described by Pereira and collaborators [37]. Quantification was performed by using particle-enhanced immunoturbidimetric methods (RF II- Tina quant RF II, COBAS, for rheumatoid factor; and CRPL3- C Reactive Protein Gen 3, COBAS, for C-reactive protein) in a COBAS INTEGRA 400 analyzer (Roche Diagnostics). Serial dilutions of each sample were prepared automatically (up to 1:128) and then, 50 μL of each sample was incubated with latex particles coated with a monoclonal anti-rheumatoid factor or anti-C-reactive protein antibodies. Agglutination, denoted by the formation of aggregates in positive samples, was determined turbidimetrically. Results are expressed as international units (IU) per milliliter (mL) and milligrams (mg) per litre (L) of a sample, for rheumatoid factor and C-reactive protein; respectively.

### 4.3. Flow Cytometry Analysis

For flow cytometry analysis, peripheral blood samples underwent red blood cell lysis. Then, single-cell suspensions were prepared and the cells were stained with Trypan blue (Sigma-Aldrich, Brazil) and assessed for viability in a haemocytometer. Cells (5 × 10^6^) were washed, resuspended in flow cytometry buffer ((2% fetal calf serum (Invitrogen, Brazil) in phosphate-buffered saline-PBS (Sigma-Aldrich, Brazil)), and stained with directly conjugated monoclonal antibodies: anti-CD14 PE (clone M5E2; BD Biosciences, Brazil), anti-HLA-DR PE-Cy5 (clone LN3; eBioscience, Brazil) and anti-CD62L FITC (clone SK11; BD Biosciences, Brazil). Events (80,000) were acquired on a BD Accuri C6 (BD Biosciences-Immunocytometry Systems) and analyzed using FlowJo software (Tree Star Inc., Ashland, OR, USA). Differential cell populations (polymorphonuclear (PMNs) and mononuclear cells) were identified by size and granularity through flow cytometry. Results are expressed as well as the number of cells per mm^3^, except for HLA-DR and CD62L, also expressed as mean fluorescence.

### 4.4. Attenuated Total Reflection-Fourier Transform Infrared Spectroscopy (ATR-FTIR)

Two µL of each plasma sample were assayed by an ATR-FTIR spectrophotometer Vertex 70 (Bruker Optics, Reinstetten, Germany) coupled with Platinum Diamond ATR and operated by an OPUS 4.0 software. Sample spectra and background were taken with 4 cm^−1^ of resolution and 32 scans were performed of each plasma sample to minimize bias. The ATR unit was cleaned and dried every time, previous to the next infrared analysis. The plasma samples were dried using airflow on ATR-crystal for 3 min before obtaining the sample spectra over the range of 4000 to 400 cm^−1^ in a room with a controlled temperature 21–23 °C [38,39].

### 4.5. Spectral Data Pre-Processing

The OPUS software was implemented for pre-processing of the original infrared spectra to normalization and baseline corrections. The second differentiation spectra were carried out using the Savitzky–Golay method in Origin 9.1 software in order to emphasize the bands, resolve overlapped bands, and enhance the precision of analysis by revealing genuine biochemical characteristics. In the smoothing pre-treatment, polynomial order of two and 20 points of the window for examination were set as parameters of the Savitzky–Golay filter, in order to find the relatively optimum smoothing effect. The second derivative generates valleys from the original absorption spectrum. Therefore, the analyzed wavenumbers in the second derivative are the height of valleys [38,40].

### 4.6. Data Analysis

Data are represented as mean + SD (VAS pain scale, total leukocytes, PMN and CD14^+^ cells, CD62L+ PMNs, and vibrational modes at 1649 cm^−1^, 2953 cm^−1^ and 3091 cm^−1^) or median and interquartile range (25–75th; IQR) or minimum-maximum values (HAQ-DI, C-reactive protein, rheumatoid factor, DAS28-ESR, SDAI, mononuclear and CD62L^+^ CD14^+^ cells, and HLA-DR and CD62L mean fluorescence in CD14^+^ cells), depending on their distribution. Parametric (one-way analysis of variance (ANOVA) followed by the Newman–Keuls’s test) or non-parametric (Kruskal–Wallis followed by the Dunn´s test) tests were used to determine the significance of differences between groups. All group comparisons and ROC curve analyses were performed in GraphPad Prism software 6.0. *p* values < 0.05 were considered statistically significant.

## 5. Conclusions

Early monitoring of treatment response in RA is pivotal to reducing unnecessary adverse effects and costs towards therapies that are not always effective. Overall, the results presented herein, indicate that both flow cytometry and ATR-FTIR can potentially be employed as additional measures to monitor treatment response to LFN in RA patients as early as three-months after treatment had started. As we performed a pilot and proof-of-concept study, further studies with a larger population that can be monitored for longer time-points to allow the full assessment of remission, are necessary in order to further validate the clinical use of ATR-FTIR and flow cytometry as measures to examine treatment response in patients with RA.

## Figures and Tables

**Table 1:** Characteristics of the patients.

Variable	Arthritis Patients		
	NST	LFN-R	LFN-NR
Age (Mean (SD))	57.5 (2.8)	57.0 (4.7)	51.8 (3.7)
Gender (number (no) of patients (%))	No (%)	No (%)	No (%)
Male	1 (7%)	01 (7%)	0 (0%)
Female	13 (93%)	07 (50.0%)	06 (43%)
Duration of disease (in years)	No (%)	No (%)	No (%)
<10	12 (86%)	08 (57%)	05 (36%)
10–19	01 (7%)	0 (0%)	01 (7%)
20–30	01 (7%)	0 (0%)	0 (0%)
Presence of swollen joints (nº of patients (%))	No (%)	No (%)	No (%)
Yes	12 (86%)	02 (14%)	06 (43%)
No	02 (14%)	06 (43%)	0 (0%)
Presence of lumps or deformities (nº of patients (%))	No (%)	No (%)	No (%)
Yes	03 (21%)	01 (7%)	01 (7%)
No	11 (79%)	07 (50%)	05 (36%)
DAS28-ESR (Median (IQR))	(6.0 (5.7–6.4))	(2.6 (2.4–2.9)) #	(6.3 (5.8–6.8))
SDAI (Median (IQR))	(50.5 (42.3–53.5))	(9.0 (4.0–11.2)) #	(53.0 (45.0–59.3))
VAS pain scale (Mean (SD))	(78.2 (11.7))	(39.4 (10.8)) #	(66.7 (5.2))
HAQ-DI (Median (IQR))	(2.7 (2.5–2.8))	(1.9 (1.8–1.9)) #	(2.5 (2.4–2.6))
C-reactive protein, mg/l (Median (IQR))	(23.1 (17.3–37.3))	(9.4 (7.2–13.5)) #	(29.4 (23.3–38.8))
Rheumatoid factor, IU/mL (Median (IQR))	(135.1 (110.2–169.8))	(89.2 (68.0–107.5)) #	(127.4 (96.8–156.5))

Patients recently diagnosed with rheumatoid arthritis (RA) but not yet receiving specific treatment with anti-rheumatic drugs, prior to leflunomide (LFN; 20 mg/day, *per os*) treatment (non-specific therapy (NST) group; *n* = 14). Responders (LFN-R) and non-responders (LFN-NR) to LFN following a three-month treatment. Continuous variables are expressed as mean (standard deviation; SD) or median (25th–75th percentile; interquartile range (IQR)). Categorical variables are summarized as n (%). DAS28-ESR: Disease Activity Score-28 for Rheumatoid Arthritis with ESR; SDAI: Simplified Disease Activity Index for Rheumatoid Arthritis; VAS: visual analog scale; HAQ-DI: Stanford Health Assessment Questionnaire-disability index, mg/mL: milligrams/litre; IU/mL: international units/milliliter. ^#^ *p* < 0.05, differs from prior to therapy (NST group).

## Data Availability

The data presented in this study are available on request from the corresponding author.
